# Maternal Exposure to Dibutyl Phthalate (DBP) or Diethylstilbestrol (DES) Leads to Long-Term Changes in Hypothalamic Gene Expression and Sexual Behavior

**DOI:** 10.3390/ijms22084163

**Published:** 2021-04-17

**Authors:** Damien Hunter, Kee Heng, Navdeep Mann, Ravinder Anand-Ivell, Richard Ivell

**Affiliations:** School of Biological Sciences, University of Adelaide, Adelaide 5005, Australia; damien.hunter@sydney.edu.au (D.H.); kee.heng.my@gmail.com (K.H.); navdeep.mann@adelaide.edu.au (N.M.); ravinder.anand-ivell@nottingham.ac.uk (R.A.-I.)

**Keywords:** hypothalamus, endocrine disruption, sexual behavior, oxytocin, kisspeptin

## Abstract

Xenobiotic exposure during pregnancy and lactation has been linked to perinatal changes in male reproductive outcomes and other endocrine parameters. This pilot study wished to assess whether brief maternal exposure of rats to xenobiotics dibutyl phthalate (DBP) or diethylstilbestrol (DES) might also cause long-term changes in hypothalamic gene expression or in reproductive behavior of the resulting offspring. Time-mated female Sprague Dawley rats were given either DBP (500 mg/kg body weight, every second day from GD14.5 to PND6), DES (125 µg/kg body weight at GD14.5 and GD16.5 only), or vehicle (*n* = 8–12 per group) and mild endocrine disruption was confirmed by monitoring postnatal anogenital distance. Hypothalamic RNA from male and female offspring at PND10, PND24 and PND90 was analyzed by qRT-PCR for expression of aromatase, oxytocin, vasopressin, ER-alpha, ER-beta, kisspeptin, and GnRH genes. Reproductive behavior was monitored in male and female offspring from PND60 to PND90. Particularly, DES treatment led to significant changes in hypothalamic gene expression, which for the oxytocin gene was still evident at PND90, as well as in sexual behavior. In conclusion, maternal xenobiotic exposure may not only alter endocrine systems in offspring but, by impacting on brain development at a critical time, can have long-term effects on male or female sexual behavior.

## 1. Introduction

Environmental endocrine disruption caused by exposure during development to common manmade chemicals can lead to several possible phenotypic outcomes. Much attention has been given to xenobiotics acting on the male reproductive system [1], largely because the testes are actively differentiating at a time during gestation when the placental barrier appears to be quite permeable, but also because the phenotypic readout of such disruption is easily visible shortly after birth (cryptorchidism, genital malformation). Other systems, however, are also likely to become disrupted due to xenobiotic exposure during development, though the phenotypic effects may be more subtle (e.g., the disruption of glucose homeostasis leading to diabetes, or of thyroid hormone metabolism during development) [2]. Moreover, partly because of this emphasis on the male reproductive phenotype, there has been a tendency to consider such xenobiotic action as having anti-androgenic or estrogenic effects, although the molecular mechanism of action for many endocrine disrupting chemicals is still largely unknown.

Diethlystilbestrol (DES) and dibutyl phthalate (DBP) are two well-characterized endocrine disrupting substances. In rodents, these compounds appear to impact target genes within Leydig cells during embryonic development, such as INSL3 [3,4] or some steroidogenic enzymes [5]. While DES, as a model estrogen, is thought to be a preferential ligand for estrogen receptor beta (ERβ, Esr2), neither DBP nor its major active metabolite monobutyl phthalate have been shown to interact directly with any steroid-activated transcription factor [6]. Both substances, however, appear to exert similar phenotypic effects on the male reproductive system [3,4]. When applied acutely and in moderate amounts to pregnant rats within the so-called masculinization window in the latter half of gestation, both compounds appear to affect fetal testis function resulting in a reduction of fetal testosterone [7,8].

The embryonic window for maximal disruptive action on the male reproductive system in rats is in the latter half of gestation, specifically GD12-GD17 [9]. This period, as well as the perinatal phase, is also a time when, particularly, the hypothalamus is developing its essential structure and functionality. It has been shown that acute maternal administration of DES to rats in this period can affect the size and gene expression within the neonatal sexually dimorphic nucleus of the preoptic area [10,11]. The nuclear structures of the hypothalamus are key components, not only for the central control of the major endocrine axes but, also, via projections to other brain regions, they are critically involved in a number of important behavioral paradigms, including maternal and sexual behavior [12,13,14]. Some of the major hypothalamic genes are moreover under direct or indirect steroid control, such as those for oxytocin (Oxt) or gonadotropin releasing-hormone (GnRH). Furthermore, the former gene is regulated by a promoter element similar to that controlling INSL3 and many steroidogenic enzymes within Leydig cells [15,16], and such genes are known to be influenced by DES and DBP in utero [3,7]. It is thus critical to consider whether experimental endocrine disrupting paradigms, such as those involving DES or DBP, may not also be impacting on hypothalamic development and function, and, consequently, on reproductive behavior.

In rodents, sex-specific differentiation of brain regions, such as the hypothalamus, is dependent on exposure to testicular-derived steroids, such as testosterone, which may be further modified by local aromatization. This results in males displaying a different anatomical phenotype to females in terms of hypothalamic nuclei size and structure [17]. A similar phenotype can be induced by perinatal dosing of female rats with natural and synthetic estrogens, such as DES [18]; this can also evoke masculinized behavior [19]. Perinatal estrogen levels may also quantitatively and qualitatively influence long-term ERα or ERβ expression in neurons [19,20,21,22,23,24,25]. Different aspects of behavior appear to be regulated by ERα and ERβ, with the former more involved in sexual behavior and the latter with spatial learning, social recognition, anxiety, and aggression [19,26,27]. Both receptors may be acting via the Oxt system, which, in rats, can facilitate some aspects of sexual behavior, including penile erection and lordosis, though also inhibiting anxiety and aggression and aiding social recognition [28,29]. In part, this anxiolytic role may be counterbalanced by effects of vasopressin (Avp), which appears to be involved in aggression and anxiety and can reduce female sexual receptivity [30]. Avp expression also seems to be regulated by ERβ, though, in males, it appears to be influenced more by androgens than by estrogens [31]. Both Avp and Oxt are thus to be considered as plausible hypothalamic targets for assessing endocrine disrupting chemical (EDC) action during development.

There is support for possible endocrine disruption of behavior by phthalates and xenoestrogens from several earlier studies on rats and mice [32,33,34]. For example, although Lee and colleagues [32] found no effect of perinatal DBP exposure on the expression of two steroid-regulated genes in the neonatal hypothalamus, they did show an effect on the lordosis quotient in the adult female offspring. The present study differs, however, in that most such studies used quite extensive dosing paradigms and assessed relatively acute responses; here, maternal xenobiotic exposure was specifically limited to only a short period within the perinatal masculinization window, and hypothalamic gene expression was measured postnatally, as well as in puberty and adulthood. Importantly, there is recent evidence to suggest that modest phthalate exposure of pregnant women is significantly associated with behavioral changes in the resulting children assessed several years later [35,36].

**Figure 1:**
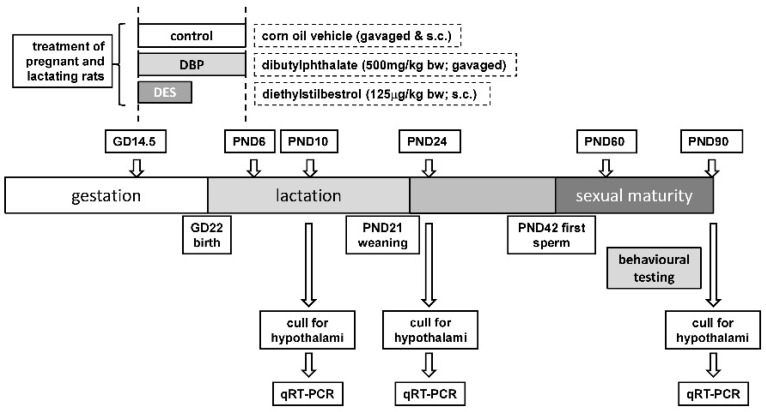
Scheme to indicate dosing and sampling procedures (for details, see text).

The main aim of this pilot study was to identify novel target endpoints in male and female offspring for maternal EDC-induced perturbation during the sensitive androgenization developmental window. It is therefore not trying to mimic conditions in humans where exposures may be prolonged and involve mixtures of low-dose xenobiotics. Rather, short exposures were selected to allow better understanding of the possible mechanisms involved. Hence, pregnant Sprague-Dawley rats were given a brief moderate dose of either DBP or DES, during a relatively short developmental window, to cause a minimal disruptive effect on the male reproductive system, though no other obvious negative health outcomes. Hypothalamic gene expression was then assessed at three time-points—before, during and after puberty—in both male and female offspring, as well as adult behavioral paradigms reflecting sexual function (Figure 1). The findings indicate subtle but persistent changes in hypothalamic gene expression, as well as long-term changes to sexual behavior.

## 2. Results

### 2.1. General Effect of Treatments

**Figure 2:**
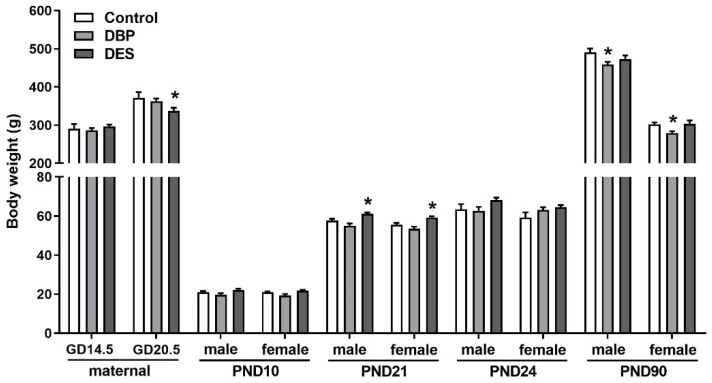
Maternal and offspring body weights in controls and rats treated maternally with either dibutyl phthalate (DBP) or diethylstilbestrol (DES) measured at the indicated gestational (GD) or postnatal days (PND). Means + SEM (*n* = 7–24 per individual category). * Indicates significant difference (*p* < 0.05) from relevant control values. Data were analyzed by ANOVA, followed by post hoc Tukey’s multiple comparison test.

Administration of DBP to pregnant and lactating rats from GD14.5 to PND6 (Figure 1) had no effects on maternal body weight or on the weights of progeny born, measured at PND10, PND21, and PND24 (Figure 2). The maternal DBP treatment resulted only in a small reduction in body weight in adulthood (Figure 2). All pregnant rats in the control and DBP groups delivered exclusively live pups; all animals appeared in good health throughout the experiment.

Administration of DES to rats at high doses has previously been associated with abortion of fetuses [37]. For this reason, DES was given to pregnant rats only on GD14.5 and GD16.5, within the masculinization window, and not at all during lactation [7,38,39]. The maternal rats showed a small acute weight loss following DES administration at GD20.5, unlike in controls and DBP-treated animals (Figure 2). Two out of the initial 12 rats in this group had still-births, whereby no live pups were born. Excluding these two rats, DES treatment led to a marginally smaller litter size (10.8 ± 1.5), compared to control (13.6 ± 1.0) or DBP (12.0 ± 0.8) groups (not significant). Once born, all pups grew well and healthily, with no differences in body weight between groups at PND10 and PND24 (Figure 2). At weaning on PND21, both male and female DES pups showed a significant (*p* < 0.05) 6–7 percent increase in body weight compared to control and DBP groups (data not shown), which, however, did not persist into adulthood (Figure 2).

### 2.2. Anogenital Distance (AGD)

**Figure 3:**
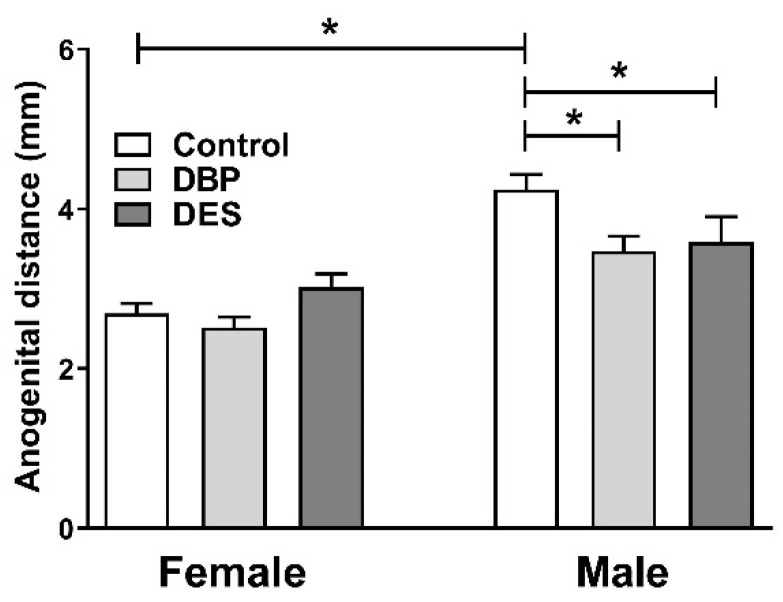
Anogenital distance measured at PND10 (means ± SEM, *n* = 11–16 per individual category) for male and female pups maternally treated with DBP or DES, compared to controls. Horizontal bars and asterisks indicate significant differences (*p* < 0.05). Data were analyzed by ANOVA, followed by post hoc Tukey’s multiple comparison test.

There were no significant between-group differences in AGD for both male (control 2.16 ± 0.50 mm) and female (control 1.16 ± 0.42) pups at PND2 (data not shown). However, at PND10, both DBP- and DES-treated male pups showed a significantly reduced AGD compared to controls (Figure 3), intermediate in size between male and female distances. Female AGD showed no effects of maternal treatment.

### 2.3. Vaginal Opening

The day of vaginal opening was also continually monitored for the female pups from PND29 as an indicator of female puberty. All treatment groups showed no effect compared to controls (day 34.5 ± 2.2; data not shown).

### 2.4. Hypothalamic Gene Expression

**Figure 4:**
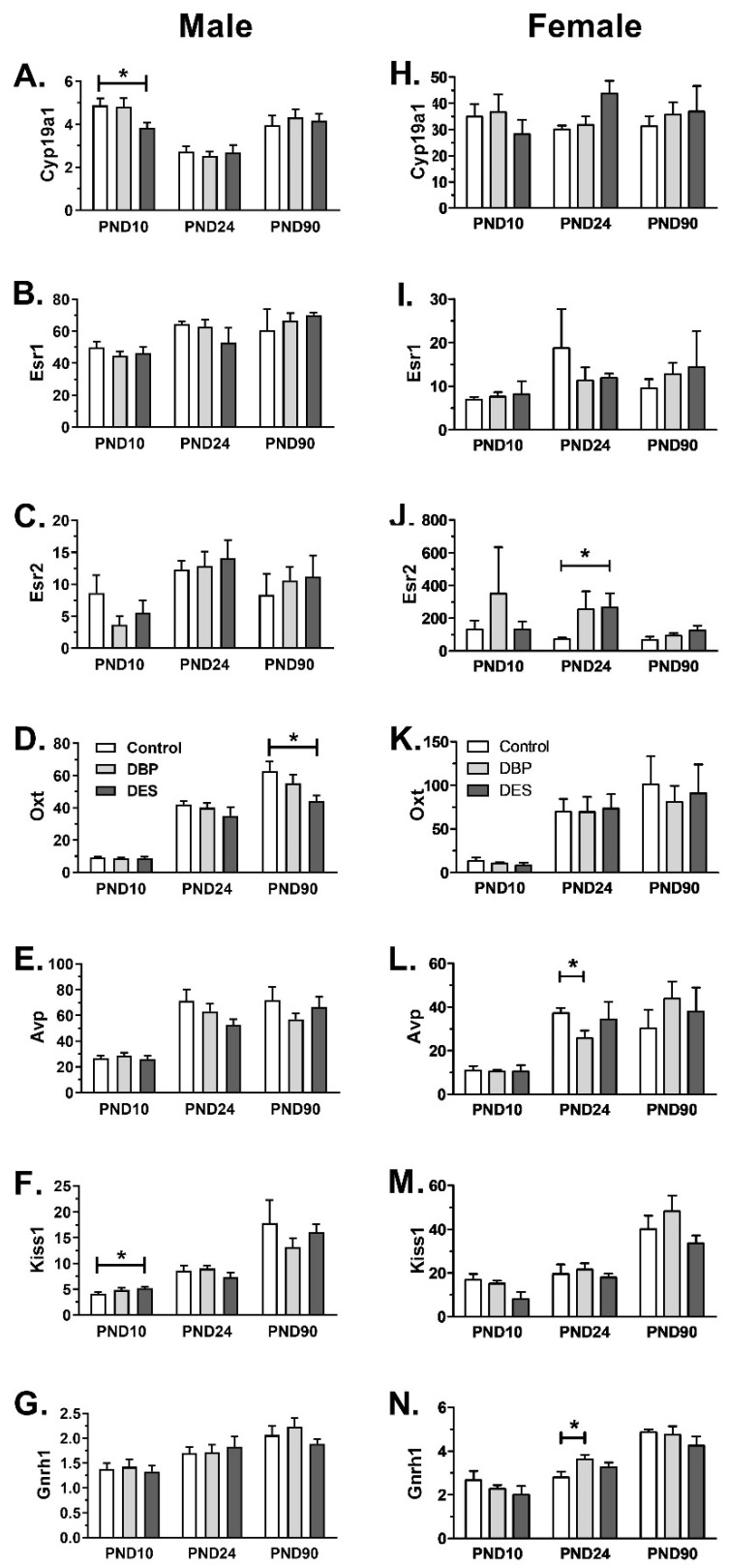
Relative transcript levels (means ± SEM, *n* = 6 per individual category) in the hypothalami of maternally treated (DBP or DES) and control (CON) rats, as indicated, determined by qRT-PCR and normalized against the housekeeping transcript for ribosomal protein S27a. Horizontal bars and asterisks indicate significant differences (*p* < 0.05). Data were analyzed by ANOVA, followed by post hoc Tukey’s multiple comparison test.

Hypothalami were carefully dissected from male and female progeny at PND10, PND24, and at the termination of the study on PND90. The tissues consistently included the major magnocellular and parvocellular nuclei of the preoptic, suprachiasmatic, supraoptic, paraventricular, periventricular, and geographically closely related regions. Gene transcripts were selected for analysis, which have been implicated in sexual or affiliative behavior (Cyp19a1, Avp, Oxt, Esr2, respectively encoding aromatase, vasopressin, oxytocin, and ERβ), in puberty onset (Kiss1, encoding kisspeptin), or in regard to regulation of the HPG axis (Esr1, Gnrh1, encoding ERα and GnRH). For both males and females, results were encouragingly consistent, with mostly small standard errors and little, if any, variance between treatments (Figure 4). This important observation reinforces the technical accuracy of the analysis and serves to emphasize the few small changes that were found to be statistically significant. For the male progeny at PND 10, Cyp19a1 gene expression (Figure 4A) was significantly reduced in the DES group compared to controls, whereas, in the same treatment group, Kiss1 gene expression was slightly but significantly elevated (Figure 4F). At later time-points, only Oxt gene expression showed a consistent effect of treatment, with DES causing a significant reduction in this specific hypothalamic mRNA (Figure 4D). In the female progeny, interestingly, quite different genes were impacted upon by the maternal xenobiotic treatment. There were no significant effects evident at PND10 but, at PND24, with the onset of female puberty, there was a marked increase in Esr2 mRNA for both DBP and DES (Figure 4J), though significance (*p* < 0.05) was only achieved for the DES group. Only DBP-treatment gave rise to a reduction in Avp gene expression at this time-point (Figure 4L), and also to an increase in specific Gnrh1 gene expression (Figure 4N). There were no significant differences in gene expression in any of the PND90 female samples.

### 2.5. Sexual Behavior

**Figure 5:**
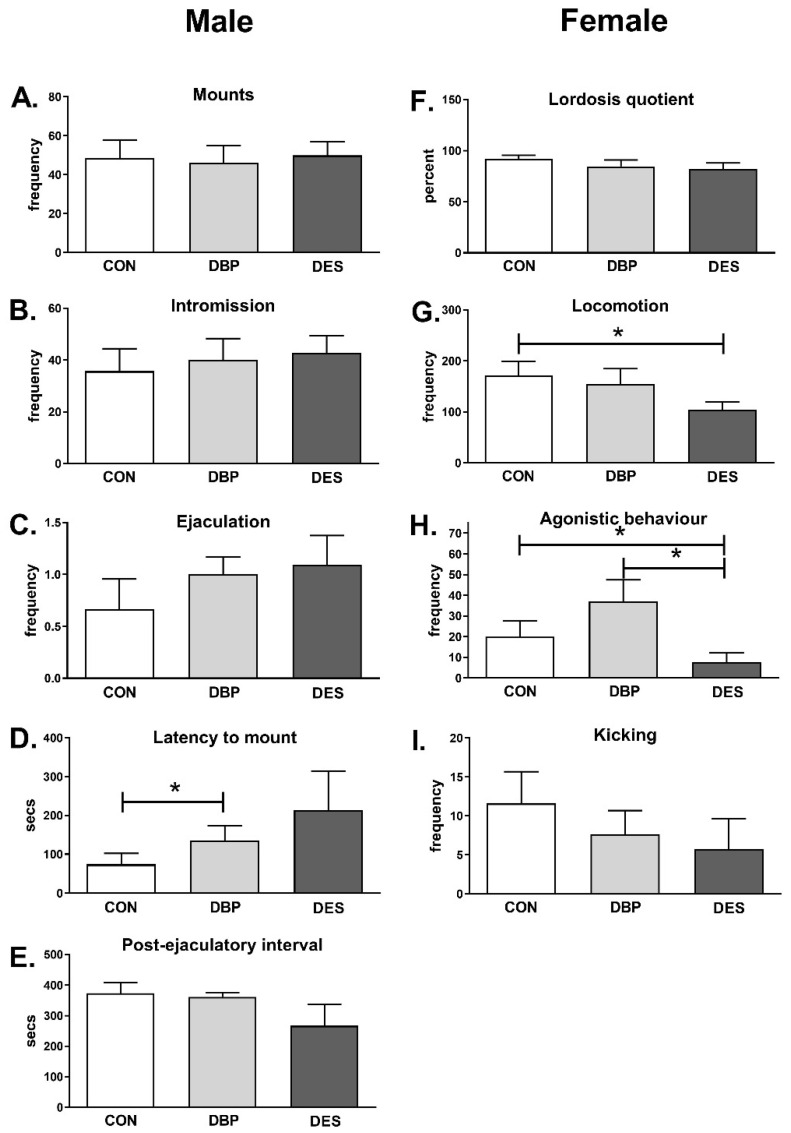
Sexual behavior (described and quantified as in Methods and Materials; means ± SEM, *n* = 8–10 per individual category) of adult male and female rats which had been treated maternally with DBP or DES. CON, controls. Horizontal bars and asterisks indicate significant differences (*p* < 0.05). Data were analyzed by ANOVA, followed by post hoc Bonferroni’s test.

Altogether, both male and female adult sexual behavior was little influenced by maternal treatments with DBP or DES (Figure 5). In the males, there was a small effect, just attaining significance, for the DBP-treatment to cause an increased latency period (Figure 5D). In the female rats, both locomotor (Figure 5G) and agonistic (Figure 5H) activities were decreased in DES-treated rats, whereas, in contrast, agonistic behavior appeared to be increased in the DBP-treated rats, though not significantly. Correlation analysis (Table 1) showed that, in all rats, there was a high correlation between lordosis and mounts. For control rats, where agonistic behavior was modest (Figure 5H), there was no relationship between this agonistic behavior and measures of sexual receptivity (i.e., mounts and lordosis posture displayed). This was quite different for DES, for which both agonistic behavior and kicking were strongly inversely correlated with sexual behavior (lordosis quotient). Indeed, kicking as a behavior was more strongly associated with agonistic behavior in the DES group than in other groups, and unlike in controls, where it strongly positively correlates with sexual receptivity. Additionally, in controls and the DES group, locomotion activity (hops and darts) was correlated positively with both mounts and lordosis posture. In contrast, in the DBP group, there was no relationship between general activity (measured by locomotion) and sexual behavior, but an inverse relationship with agonistic behavior. That is, rats which spent less time in sexually related locomotion (hops and darts) displayed more aggressive behavior. In summary, controls and maternally DBP-treated females appear to remain sexually receptive, regardless of any aggression; in contrast, the maternal DES treatment lead to a segregation of aggressive behavior at the cost of sexual receptiveness.

## 3. Discussion

We have chosen, here, a dosing regimen for both DES and DBP which had, as primary objective, sufficient xenobiotic exposure to induce minimal but significant disruption of the classic androgenization endpoint of a reduced AGD in male pups, though without any obvious or significant effects on general health parameters. Moreover, the DES treatment was additionally restricted to the prenatal period since it has been shown that longer or later exposures had more complex and severe repercussions [40]. Thus, any changes observed in specific parameters are not attributable to a general health deficit but should be due to a specific disruption of those features. These criteria were met and strongly supported by the generally low within- and between-group variance in most parameters.

Within the dissected hypothalamus, which includes several important functional nuclei, some changes may be masked by the complexity of the tissue, especially where individual genes are expressed in more than one type of nucleus (e.g., Avp or Oxt). Thus, any changes observed are more likely to be physiologically significant as, for example, the effect of osmotic stress on Avp and Oxt expression [41]. Changes in gene expression were evident for both male and female offspring. In the males, whereas the changes in Cyp19a1 and Kiss1, only evident at PND10, and only for DES treatment, are likely acute responses to estrogenic treatment, the 30% drop in Oxt mRNA in adult males and a lesser, though not significant, reduction in the DBP treatment group for the same gene transcripts, is evidence of a persistent effect on a known estrogen-responsive gene, with documented effects on a variety of behavioral paradigms in mammals, including affiliative and aggressive behaviors, maternal behavior, anxiety, sexual function, and appetite [28,42,43]. Interestingly, for the female hypothalami, variance for most, though not all, parameters was higher, possibly reflecting nascent hormone cyclicity, with significant treatment-dependent effects only evident at PND24, 18 days following cessation of DBP exposure and longer after cessation of DES treatment. At this time point, both treatments led to an increase in Esr2 gene expression (significant only for DES), a reduction in Avp mRNA, and an increase in Gnrh1 mRNA, both significant only for the DBP treatment. None of these effects persisted into adulthood. PND24 is a time-point early in the onset of puberty in rats when the HPG and HPA axes become activated, and many hormone systems are in a highly dynamic state. By PND90, most hormonal systems will have acquired their final adult quantitative parameters. These results imply that DBP or DES may have acute effects, or effects influencing the rate of establishment of various hormone systems; but only for one of the genes tested, Oxt in the male, is there evidence for a longer term induced change impacting on hypothalamic “hard-wiring” or programmed status.

All behavioral tests were carried out on adult animals (PND60-PND90) whose hypothalamic gene expression is represented by the PND90 values. Moreover, it is important to note that male sexual behavior was analyzed using intact female stimuli rather than ovariectomized and hormonally primed females, the former possibly eliciting a greater variance in male response. Interestingly, for both males and females, there was minimal effect of both interventions on sexual activity, as reflected by mounting, intromission and ejaculatory frequencies in the males, and by lordosis quotient in the females (Figure 5). In males, there is a marked impact on latency to mount for both DBP and DES treatment groups (though only significant for the DBP group). This is interesting because penile erection in rats has been shown to be linked to hypothalamic oxytocin expression [33,44], suggesting that latency in this case might be due to a transient erectile dysfunction. In females, sexual activity is accompanied by a series of stereotypical behavior patterns, including the well-known post-copulatory hops and darts (here referred to as “locomotion”), as well as sexually related kicks [45]. It is interesting to note that, while we have no obvious changes in the hypothalamic expression of the gene transcripts examined in the female rats, xenobiotic treatment appears to influence particularly the kicking and agonistic behavior associated with sexual activity (boxing, frontal and lateral threats, biting), which is reduced with maternal DES treatment. DBP treatment has no significant effect on any female behavioral parameter per se, though the correlation analysis (Table 1) does suggest a significant negative relationship between agonistic behavior and the classic sexually related locomotory activity of hops and darts (locomotion). This analysis also reveals that the standard sex-related agonistic activity (kicking) seen in the controls, and significantly associated with mounts and lordosis, loses that relationship in the offspring maternally treated with DBP or DES, with the latter group even showing a significant negative correlation between these behavior paradigms. This suggests that a more generalized aggression, when it occurs, is quite independent of sexual activity.

In this study, a maternal dosing regimen of two xenobiotics, DES and DBP, was chosen in order to provide a minimal, though significant, endocrine disruptive effect on male reproductive endpoints, though otherwise having little effect on alternative parameters (e.g., vaginal opening), or on general health. In regard to the male reproductive phenotype, for example, cryptorchidism or fetal androgen production, both compounds appear to lead to similar disrupted phenotypes [2], although their possible molecular mechanisms of action are likely to be quite different. Here, we have extended this experimental paradigm to assess the hypothalamus, via limited study of gene expression, as well as to look at behavior which is known to be influenced by hypothalamic input. The hypothalamus is still relatively plastic at this developmental time-point, and is therefore vulnerable in regard to exogenous manipulation of the steroidal milieu; moreover, much sex-specific behavior is regulated via hypothalamic function. Our results suggest that, in regard to hypothalamic gene expression and to behavior, both DBP and DES have effects, though, for some parameters, there is insufficient power to resolve all aspects. In male latency to mount, DBP and DES appear to behave in a similar way by increasing the delay before mounting. In contrast, in females, DES appears to cause a reduction in agonistic behavior, as well as sexually related locomotion; but, in doing so, establishes a significant negative correlation between agonistic behavior, kicking and sexual receptivity. DBP appears to be different in that it has little effect on female sexual behavior, except to remove the positive correlations seen in controls between lordosis, mounts, kicking and locomotion, suggesting a dysregulation of the coordination between these sexual paradigms. Obviously, more detail through a larger study is needed in order to explore precisely the mechanisms by which these long-term changes are achieved. Nevertheless, this preliminary study, together with studies on children and their mothers in the USA [35,36], highlight the possibility that maternal exposure to endocrine disrupting substances can influence not only endocrine parameters but also affect sex-specific behavioral paradigms. Future research will require a much more detailed analysis of both gene and protein expression in the brain, as well as behavior and gene epigenetic status so that these can be sufficiently correlated to suggest the causal mechanisms involved.

Dosing regimens have been used in this study which are considerably in excess of normal human exposures. However, direct comparisons are spurious since humans are exposed to a multiplicity of agents which may act synergistically, for more extensive periods, and may reflect species-specific differences in their impact [2]. Even low symptom penetrance in the human population can be medically significant. Latest EDC research suggests that the concept of safe levels, represented experimentally as LOAELs (lowest adverse effect level) or NOAELs (no adverse effect level), and defining safety thresholds, may not be theoretically valid [46,47]. Such thresholds are determined using model animals and systems which do not account for all human exposure scenarios, nor do they take account of non-monotonic dose-responsiveness [47], implying that a dose-independent hazard assessment concept, as is applied to cancer-causing agents, may be more appropriate in considering EDC action [46].

## 4. Materials and Methods

### 4.1. Ethics Statement

All animal experimentation was approved by the University of Adelaide Animal Ethics Committee (approval S-019-2007).

### 4.2. Animals and Treatments

Nine-week old female Sprague-Dawley rats from Laboratory Animal Services (University of Adelaide) were mated overnight and checked the next morning for the presence of sperm in vaginal smears. The morning when sperm were observed in the vaginal smears was designated as gestation day 0.5 (GD0.5). The day when the pups were delivered was designated as postnatal day 1 (PND1). Pregnant rats and their offspring were maintained on soy-free standard AIN93G rodent diet (Specialty Feeds, Western Australia, Australia) throughout the experiment. Pregnant rats were divided into 3 groups, using randomization of body weights to ensure equal weight distribution among groups, and dosed as following: Control group (*n* = 8): orally gavaged with corn oil (Sigma-Aldrich, North Ryde, NSW, Australia; #C8267) 1 mL/kg body weight every second day from GD14.5 to PND6 and subcutaneously (s.c.) injected with corn oil containing 0.5% ethanol on GD14.5 and GD16.5. DBP group (*n* = 9): orally gavaged with DBP (Sigma-Aldrich; #524980, >99% pure) 500 mg/kg body weight in corn oil every second day from GD14.5 to PND6 and s.c. injected with corn oil containing 0.5% ethanol on GD14.5 and GD16.5. DES group (*n* = 12): orally gavaged with corn oil 1 mL/kg body weight every second day from GD14.5 to PND6 and s.c. injected with DES (Sigma-Aldrich; #D4628, >99% pure) 125 µg/kg body weight in corn oil containing 0.5% ethanol on GD14.5 and GD16.5 only. The doses of DES and DBP used were based on the mid-range effective values from previous studies (e.g., [7,9,40,48,49]). Whilst likely giving rise to circulating levels substantially higher than those measured for equivalent substances in the human population, these limited doses (which are less than the ED50 for impacts on male physiology in the rat and are reported to have no immediate impact on female physiology [50,51]) appear to indicate appropriate phenotypes in rats with high penetrance, and low general toxicity, allowing relatively small group numbers in accord with 3R principles. The doses are moderately greater than the latest LOAELs reported for DBP (2 mg/kg/d) or DES (0.5 µg/kg/d) in the context of reprotoxic effects in rats [52,53], with a tolerable daily intake dose for DBP in the human population of 0.01 mg/kg/d [52], and which are used to guide regulatory decision-making. On PND2, excess pups were culled, retaining 10 pups (female:male ratio ~1:1) per litter. On PND21, both male and female pups were weaned and caged with their littermates of the same gender. Male and female progeny were culled by CO_2_ asphyxiation as indicated on PND10, 24 and 90 (Figure 1).

### 4.3. Measurement of Anogenital Distance (AGD)

The anogenital distance (AGD) was measured from the base of the genitals to the center of the anal opening for all pups before culling on PND2 and PND10. Pups were digitally photographed from the ventral aspect, including a precise 1 mm scale in each picture as reference point. All images were imported to the same computer and monitor for all measurements. These were repeated twice several days apart by the same observer, who was blinded to the treatment identities. The AGD for each animal was the average of the two values obtained from the repeat measurements.

### 4.4. Vaginal Opening in Female Offspring

Female offspring were checked daily from PND29 onwards for the onset of sexual maturity, as determined by the separation of the vaginal membrane (i.e., vaginal opening).

### 4.5. Hypothalamic mRNA Analysis

Immediately after culling, hypothalami were cleanly dissected from the ventral aspect of the brain using 2 mm anterior to the optic chiasm as anterior margin, the posterior edge of the mammillary bodies as posterior margin, the lateral sulci as lateral margins, and dissecting dorsally to a depth of 4 mm. Thus, the principal hypothalamic magnocellular and parvocellular nuclei were included. RNA was extracted from total hypothalami using the TRIzol reagent (Invitrogen, Melbourne, Australia) and quality checked by electrophoresis. For 6–7 animals per experimental group, 3 µg total RNA per sample was reverse transcribed using Superscript II (Invitrogen) reverse transcriptase primed by oligo(dT) according to the manufacturer’s instructions.

Quantitative RT-PCR (qRT-PCR) was carried out as previously described [54] using a Rotor-Gene 3000 (Corbett Research/Qiagen, Chadstone VIC, Australia) real-time PCR cycler, using Sybr-Green pre-mix (Takara, Shiga, Japan), with oligonucleotide primers and PCR conditions as listed in Table 2. Primer pairs were designed using Primer 3 software to span at least one intron. Transcript levels were normalized against the ribosomal protein transcript Rps27a [55]. This reference transcript had previously been found by microarray analysis to vary least between different tissue type and treatments [55]; in the present study, it was also shown by qRT-PCR, without normalization and expressed only as a function of input RNA, to be highly consistent (overall coefficient of variation, 1.8%, *n* = 55), with no significant variation between preparations irrespective of sex, age and/or treatment (data not shown). For each PCR reaction, melt curves were calculated of final products, and each was subsequently checked by individual agarose gel electrophoresis for correct size, followed by sequencing.

### 4.6. Behavioral Testing

Sexual behavior testing, modified from Lee et al. [32] and Pedersen and Boccia [30], was carried out on PND60-PND90 rats between 11.00 p.m. and 5.00 a.m., with all tests being video-filmed using Microsoft Life-Cam VX-3000 cameras under red light. Sexual behavior testing was aided by mirrors so that animals were visible from all angles. Rats were placed in a clear Plexiglas box with either a highly estrous proven breeder (for males; *n* = 9 per treatment group) or sexually experienced vasectomized male (for females; *n* = 10 per treatment group). For males, each 30-min trial was video-recorded and later frequency and latency to mount, intromission, ejaculation and post-ejaculatory interval (PEI) were measured. For females, the lordosis quotient, sexually-related locomotion (hops and darts), kicking, and agonistic behavior (boxing, defensive, frontal and lateral threat, biting) were measured, with variables as defined by Pedersen and Boccia [30], and with lordosis defined as 2–3 points on Hardy and Debold’s lordosis scale [45]. Because of possible treatment effects, estrus was not induced in the females; instead, all trials were repeated daily until the female cycled naturally into estrus. Only results from that day were included in the analyses.

### 4.7. Statistical Treatment

Graphpad Prism software was used for all statistics. Comparisons between body weight, AGD and gene expression in control, DBP and DES groups of each sex were made using one-way ANOVA, comparing groups by Tukey’s multiple comparison test. One-way ANOVA with post-hoc Bonferroni tests were used to compare behavioral data between sexes and treatments. Bivariate correlations (Pearson’s r) were used to examine relationships between female sexual behavioral variables. In all tests, alpha significance was considered to be *p* ≤ 0.05.

## Figures and Tables

**Table 1:** Correlation coefficients (Pearson’s r) for female sexual behavior patterns. (* indicates statistically significant correlations, *p* < 0.05).

	Mounts	Lordosis	LQ	Locomotion	Kicking	Agonistic Behavior
**Mounts**						
CON		0.998 *	0.401	0.734 *	0.699 *	0.032
DBP		0.936 *	0.542	0.353	−0.353	−0.423
DES		0.977 *	0.238	0.731 *	−0.337	−0.388
**Lordosis**						
CON			0.426	0.749 *	0.674 *	0.005
DBP			0.385	0.145	−0.467	−0.452
DES			0.411	0.789 *	−0.417	−0.465
**LQ**						
CON				0.598	0.151	0.051
DBP				0.475	−0.183	−0.319
DES				0.566	−0.825 *	−0.821 *
**Locomotion**						
CON					0.414	−0.107
DBP					−0.463	−0.757 *
DES					−0.556	−0.535
**Kicking**						
CON						0.527
DBP						0.538
DES						0.991 *

**Table 2:** PCR primers and conditions.

Name	Accession Number	Forward (5′–3′)	Reverse (5′–3′)	Size (bp)	Annealing Temp. (°C)
Cyp19a1	NM_017085	cccctggacgaaagttctattg	cagcgaaaatcaaatcagttgc	238	60
Esr1	NM_012689	gcgcaagtgttacgaagtgg	aagcctggcactctctttgc	375	68
Esr2	NM_012754	ctcctttagcgacccattgc	cctggatccacacttgacca	401	68
Oxt	NM_012996	ctggatatgcgcaagtgtcttc	gaaggaagcgccctaaaggtat	310	64
Avp	NM_016992	gctacttccagaactgcccaag	cagccagctgtaccagcctaa	393	64
Kiss1	NM_181692	cagctgctgcttctcctctg	ggcttgctctctgcataccg	152	62
Gnrh1	NM_012767	gaacttcgaatgcactgtccac	gctgggtatcgaaatgcggaag	211	60
Rps27a	NM_031113	ccaggataaggaaggaattcctcctg	ccagcaccacattcatcagaagg	297	64

## Data Availability

The data presented in this study are available on request from the corresponding author.
